# Supplementary material for: Collaborative intelligence in AI: Evaluating the performance of a council of AIs on the USMLE
Source: PLOS Digit Health. 2025 Oct 9;4(10):e0000787. doi: 10.1371/journal.pdig.0000787 (PMC12510544; doi:10.1371/journal.pdig.0000787)
Supplement: S2 File — (ZIP) [file pdig.0000787.s002.zip › S2 Data Raw data files containing unprocessed question inputs and LLM outputs including transcripts of deliberations/STEP3_Question-106-1_Type-MC-NJ.docx]

**Facilitator** **[Round 1]:** A 47-year-old man is admitted to the hospital through the emergency department because of the sudden onset of palpitations, left-sided chest pain, light-headedness, and shortness of breath that began while he was watching television 2 hours ago. Medical history is remarkable for paroxysmal atrial fibrillation. The patient is 180 cm (5 ft 11 in) tall and weighs 82 kg (180 lb); BMI is 25 kg/m². ECG obtained in the emergency department showed atrial fibrillation with narrow QRS complex. Pulse was 146/min. Physical examination was remarkable for rapid heart rate. The patient was given oxygen via nasal cannula and intravenous metoprolol 5 mg every 5 minutes for a total of 15 mg. His pulse slowed to 90/min. Two hours after admission, he is pain free but his pulse is now 160/min. Blood pressure is 122/78 mm Hg. In order to decrease the patient's pulse, which of the following should be administered intravenously? (A) Adenosine (B) Digoxin (C) Enalapril (D) Lidocaine (E) Metoprolol

**AI_D:** RESTATING THE QUESTION: A 47-year-old male patient who has a history of paroxysmal atrial fibrillation is admitted due to sudden onset of palpitations, chest pain, and breathlessness. He was previously administered metoprolol intravenously which initially controlled his pulse rate, but his pulse has since increased again to an elevated 160 beats per minute. The question is asking what medication should be given intravenously to once more lower the patient's pulse rate. The options are adenosine, digoxin, enalapril, lidocaine, and metoprolol. PLANNING: The assumption is that the patient needs another drug administration to lower his heart rate. This could be either a repeat dose of metoprolol or a different drug. However, we should review this assumption and contemplate whether metoprolol, which was initially effective, would still be the best option now that the patient's heart rate has risen again. We also need to consider the specific action of each drug to determine which would be most appropriate. CLINICAL REASONING: In this case, metoprolol—a beta-blocker—was administered when the patient first arrived and initially showed positive effects by lowering the patient's heart rate. The increased pulse rate after the decrease indicates that additional measures might be needed to control it. Digoxin can lower heart rate by increasing parasympathetic activity, but its onset of action is slow, so it may not be ideal for acute management. Enalapril, an ACE inhibitor, is primarily used for lowering blood pressure and does not have a substantial effect on heart rate. Lidocaine is a sodium channel blocker commonly used for ventricular arrhythmias and does not significantly impact the atrial rate. Adenosine is used in diagnosing supra-ventricular tachycardias rather than in managing atrial fibrillation. CONCLUDING: After taking into consideration the relevant pharmacology and the clinical context, metoprolol seems the best choice. Its previous effectiveness supports its selection, and re-administering it could provide the necessary rate control. RESPONSE: The optimal drug to administer intravenously in order to decrease the patient's pulse would be (E) Metoprolol.

**AI_E:** (1) RESTATING THE QUESTION: A 47-year-old man presented with rapidly changing heart rate (pulse), from a baseline of atrial fibrillation (a type of irregular heartbeat). He was initially managed with metoprolol - a beta blocker that slows the heart rate. After a brief period of successful heart rate control, his pulse again escalated, necessitating consideration of further treatment options. These include adenosine (a drug which can "reset" the heart's electrical activity), digoxin (another heart rate slowing medication), enalapril (an ACE inhibitor, primarily used for blood pressure control), lidocaine (an anti-arrhythmic used mainly for ventricular arrhythmias), or additional metoprolol. (2) PLANNING: The underlying assumption is that the patient's primary issue is rate control due to his atrial fibrillation. Another assumption could be that he is not responding to metoprolol. We should evaluate whether metoprolol is still the most suitable treatment option in this scenario, given its initial efficacy followed by lack of sustained control. Alternatively, could his heart rate be managed with one of the other listed therapeutic options? (3) CLINICAL REASONING: The patient's response, or lack thereof, to the metoprolol is a critical point. Two hours after receiving metoprolol, he had a significant increase in heart rate again. It suggests he may require another dose or another drug to control the heart rate. The choice of drug should take into account this patient's clinical scenario: he was initially responsive to metoprolol, implying a likely primary aberration in his cardiac rate rather than rhythm. Enalapril and lidocaine do not directly decrease heart rate like metoprolol, eliminating them as options. Adenosine could be handy in some cases of supraventricular tachycardia, but may not provide the sustained response needed here. Digoxin slows down heart rate, but slower onset of action and its narrow therapeutic index make it less suitable in this acute setting. (4) CONCLUDING: Although there was a concern about potential metoprolol insensitivity, it remains the most suitable option for acute control of this patient's heart rate amongst those provided. The temporary heart rate control achieved following initial metoprolol administration supports this conclusion. (5) RESPONSE: Additional metoprolol (Option E) should be administered intravenously to this patient to decrease his pulse.

**AI_C:** RESTATING THE QUESTION: The scenario describes a 47-year-old man with a sudden onset of cardiovascular symptoms, including a rapid heart rate, likely due to paroxysmal atrial fibrillation, according to his medical history. After receiving oxygen and intravenous metoprolol, his pulse rate decreased, but quickly rose again to 160/min even though the pain was gone. The query revolves around the appropriate intravenous medication that should be administered to reduce the patient's pulse rate. PLANNING: The presented question essentially pertains to understanding various drugs used to control pulse rate in a case of atrial fibrillation. Metoprolol, a beta-adrenergic blocker, has already been provided to him with transient improvement, but the pulse rate has since increased again. The other options presented include adenosine, digoxin, enalapril, and lidocaine. To come to an informed answer it is important to understand each drug's kind of action, its use in controlling heart rate, and whether it's suitable given the patient's condition. CLINICAL REASONING: Metoprolol works by reducing the heart rate, the force of heart muscle contraction, and blood pressure, thus lessening the cardiac workload. Given that this patient's pulse rate increased again following the administration of metoprolol suggests the need for an additional or alternative approach. Adenosine is used in treating supraventricular tachycardia, but is not ideal for atrial fibrillation. Digoxin reduces the rate of electrical conduction through the AV node and hence reduces heart rate - making it a plausible choice in this context. Enalapril is an angiotensin-converting enzyme (ACE) inhibitor used to treat high blood pressure and heart failure, but its effect on heart rate is indirect and not immediate. Lidocaine, a local anesthetic and anti-arrhythmic, is not typically administered to reduce heart rate, especially in the context of atrial fibrillation. CONCLUDING: Based on the clinical reasoning and understanding of the pharmacological aspects of the given options, digoxin appears to be the most viable option for this patient. Digoxin effectively decreases the heart rate in atrial fibrillation due to its action on AV nodal conduction. RESPONSE: Therefore, in order to reduce this patient's pulse rate, Digoxin (B) should be administered intravenously.

**AI_A:** RESTATING THE QUESTION: The patient, a 47-year-old man experiencing a rapid heartbeat due to a recent episode of paroxysmal atrial fibrillation, was admitted to the hospital. His heart rate decreased after the administration of metoprolol but later increased again. A treatment option is now being sought to control the recurrent rapid pulse. PLANNING: The main assumption here is the correct choice of medication to manage this patient's tachycardia (rapid heart rate). The alternatives are Adenosine, Digoxin, Enalapril, Lidocaine, and Metoprolol. Each of these drugs functions differently. Metoprolol, a beta-blocker, slows the heart rate and is often the first choice for controlling atrial fibrillation. Digoxin is used less often because it's not as effective in controlling heart rate, especially during activity. Adenosine, is a fast-acting drug that is typically used for supraventricular tachycardia but usually not in atrial fibrillation. Lidocaine is typically used for ventricular arrhythmias. Enalapril is an ACE inhibitor, typically used for hypertension and heart failure. Given the patient's condition, the ideal drug would be Metoprolol as it has been shown to successfully decrease heart rate in atrial fibrillation. CLINICAL REASONING: This patient's primary issue is the sudden onset of a high heart rate. The heart rate had initially responded to metoprolol, a beta-blocker, before increasing again. This implies his atrial fibrillation is not effectively controlled, making a repeat dose of metoprolol the most likely candidate as it had a proven earlier effect and beta-blockers are commonly used in this scenario. Other drugs like adenosine don't typically target atrial fibrillation. Lidocaine primarily targets ventricular tachycardias, not atrial. Digoxin, though used for heart rate control, has more side effects and is usually reserved for those who are not as active or have heart failure along with atrial fibrillation. Enalapril, while beneficial for conditions such as heart failure or hypertension, wouldn't play a primary role in managing acute symptomatology. CONCLUDING: Based on the clinical scenario and comparative analysis of the possible medications, metoprolol is the most appropriate choice for this patient. It's specifically indicated for rate control in atrial fibrillation, and the patient has already shown a positive response to this medication, although, it was not sustained, potentially due to the paroxysmal nature of his atrial fibrillation. RESPONSE: The most effective intravenous medication to administer to this patient to decrease his pulse would be (E) Metoprolol.

**AI_B:** (1) RESTATING THE QUESTION: The scenario elucidates a 47-year-old man who presented with palpitations, chest pain, light-headedness, and shortness of breath. His medical history is notable for paroxysmal atrial fibrillation, a condition where the heart beats irregularly and often rapidly. He was admitted to the hospital where his ECG confirmed atrial fibrillation and his pulse was found to be unusually high. He was treated with oxygen and metoprolol, a type of beta blocker that reduces heart rate. Although his pulse initially slowed, it increased again two hours post admission. The question posed is which medication can be administered intravenously to decrease his pulse. (2) PLANNING: It seems reasonable to presume that due to the patient's history of paroxysmal atrial fibrillation, his current symptoms and raised pulse rate are likely due to this condition. The increase in his heart rate after his pulse initially slowed with metoprolol raises the possibility that his atrial fibrillation is not being well controlled. However, we need to consider the properties of the medications listed in the response options. Adenosine, Digoxin, Enalapril, and Lidocaine are all important cardiovascular medications but they act via different mechanisms. Adenosine is primarily used to terminate supraventricular tachycardia but is not typically used for atrial fibrillation as it primarily acts on the atrioventricular node. Digoxin, on the other hand, is effective for controlling the ventricular rate in atrial fibrillation. Enalapril is an ACE inhibitor used mainly in hypertension and heart failure but would not directly reduce heart rate. Lidocaine is used for ventricular arrhythmias and is unlikely to be effective in atrial fibrillation. More metoprolol might be a reasonable choice given its effectiveness in slowing the heart rate. (3) CLINICAL REASONING: The most plausible route to pursue to control this patient's elevated heart rate is to continue efforts to control his atrial fibrillation, as this seems to be the underlying driver of the problem. Among the medications listed, both adenosine and lidocaine can be ruled out because they are not typically used in the management of atrial fibrillation. While enalapril may improve heart function, it doesn't directly decrease heart rate. Digoxin, although it is a traditional drug used in managing atrial fibrillation, it has slower onset and often requires several hours to start showing effect. Metoprolol, as a beta-blocker, is a first-line therapy for atrial fibrillation and is particularly useful for ventricular rate control. The patient responded initially to the metoprolol, so it seems reasonable to try administering more to try to further control the heart rate. (4) CONCLUDING: Given the acute increase in heart rate in a patient with known atrial fibrillation who initially responded to metoprolol, the priority should be to administer more metoprolol to further control the heart rate. The other medications listed, while they do have cardiovascular effects, are not the best options in this specific clinical scenario. (5) RESPONSE: Therefore, to decrease this patient's pulse, (E) Metoprolol should be administered intravenously.

**Facilitator** **[Round 2]:** Summary: AI_A and AI_B both agree that Metoprolol is the best choice for the patient. They argue that Metoprolol, a beta-blocker, is the first choice for controlling atrial fibrillation and the patient had initially responded to it. They suggest that the patient's heart rate increase might be due to the paroxysmal nature of his atrial fibrillation, and therefore, a repeat dose of Metoprolol is the most likely candidate. AI_C, however, suggests that Digoxin should be administered. They argue that Digoxin effectively decreases the heart rate in atrial fibrillation due to its action on AV nodal conduction. They believe that the patient's pulse rate increase following the administration of Metoprolol suggests the need for an alternative approach. AI_D and AI_E also agree with AI_A and AI_B that Metoprolol is the best choice. They argue that Metoprolol was initially effective in lowering the patient's heart rate, and re-administering it could provide the necessary rate control. Key Differences: The key difference in the perspectives lies in the choice of medication for the patient. AI_A, AI_B, AI_D, and AI_E believe that Metoprolol is the best choice due to its initial effectiveness and its common use in controlling atrial fibrillation. AI_C, however, believes that Digoxin is the best choice due to its action on AV nodal conduction and the need for an alternative approach after the patient's pulse rate increased following the administration of Metoprolol. Question for Council Members: Could you elaborate on why you believe Metoprolol/Digoxin is the best choice for the patient, considering the patient's pulse rate increase following the administration of Metoprolol?. Based on your preceding response, how would you answer the original question: [A 47-year-old man is admitted to the hospital through the emergency department because of the sudden onset of palpitations, left-sided chest pain, light-headedness, and shortness of breath that began while he was watching television 2 hours ago. Medical history is remarkable for paroxysmal atrial fibrillation. The patient is 180 cm (5 ft 11 in) tall and weighs 82 kg (180 lb); BMI is 25 kg/m². ECG obtained in the emergency department showed atrial fibrillation with narrow QRS complex. Pulse was 146/min. Physical examination was remarkable for rapid heart rate. The patient was given oxygen via nasal cannula and intravenous metoprolol 5 mg every 5 minutes for a total of 15 mg. His pulse slowed to 90/min. Two hours after admission, he is pain free but his pulse is now 160/min. Blood pressure is 122/78 mm Hg. In order to decrease the patient's pulse, which of the following should be administered intravenously? (A) Adenosine (B) Digoxin (C) Enalapril (D) Lidocaine (E) Metoprolol]

**AI_E:** Restatement: The key question is: What is the best intravenous treatment to lower a 47-year-old man's rapid pulse caused by paroxysmal atrial fibrillation, which has not stayed under control after an initial Metoprolol administration? Planning: The assumption here is that Metoprolol, due to its initial effective response, should be re-administered to lower the increased pulse rate of the patient in atrial fibrillation. Alternatively, Digoxin might be used, due to its potent effect on AV nodal conduction. A third alternative could be the usage of Adenosine, which can be used to manage supraventricular tachycardia by blocking the transmission of electrical impulses through the AV node. Key factors to consider are the potential side effects and efficacy of each medication, the patient's past and current medical history, symptoms, response to previous medication, and other relevant physiological or pathophysiological processes. Clinical reasoning: Metoprolol, as a beta-blocker previously used for this patient, has been successful initially in controlling the patient's atrial fibrillation and decreasing the heart rate. However, the paroxysmal nature of the patient's atrial fibrillation could have caused the increased heart rate 2 hours post initial treatment, suggesting that it might be valuable to administer a second dosage. Conclusion: However, repeated dosage of Metoprolol must be carefully evaluated due to potential side effects such as hypotension, heart block and bronchospasm. On the other hand, Digoxin, a cardiac glycoside known for its rate control in atrial fibrillation, could be an alternative, as it slows AV nodal conduction and increases parasympathetic activity, thus decreasing the heart rate. However, Digoxin requires close monitoring due to its narrow therapeutic window and potential for toxicity. Response: Weighing all the information and considering the patient's initial positive response to Metoprolol, and the need to quickly stabilize the patient's heart rate, I would choose to readminister Metoprolol (E). It has proven effective for this patient and, with careful monitoring, could again provide the necessary rate control. This recommendation is based on the information provided, and the decision should ultimately be made in a real-life situation by a qualified healthcare professional familiar with the patient's condition and medical history.

**AI_D:** RESTATING THE QUESTION: The issue at hand is deciding on the best course of treatment to reduce a middle-aged man's heart rate, who has a notable medical history of paroxysmal atrial fibrillation, after Metoprolol initially slowed his pulse but then it increased again. We are specifically deciding between Adenosine, Digoxin, Enalapril, Lidocaine, or another dose of Metoprolol. PLANNING: My reasoning is based on the understanding that the patient's atrial fibrillation is paroxysmal, meaning it can start and stop on its own, the presence of a narrow QRS complex which is a common feature of atrial fibrillation, and the patient's responsiveness to Metoprolol initially. Also, it's important to consider that the effective drug should resynchronize the AV nodal conduction and reduce the heart rate without causing drastic BP changes. CLINICAL REASONING: Metoprolol, a beta-blocker, initially lowered the patient's heart rate, indicating responsiveness. The increase in pulse could indeed be related to the paroxysmal nature of his atrial fibrillation rather than drug failure. Atrial fibrillation can cause rapid heart rates that start and stop suddenly, which seems consistent with the patient's case. It's also worth noting that beta-blockers like Metoprolol are primary treatment options for atrial fibrillation due to their effectiveness in rate control by blocking the influence of adrenaline (which can provoke a racing heart rate) in the heart. CONCLUDING: While Digoxin can also reduce heart rates, it typically works more slowly and might not be as effective in emergent situations, also its use had been associated recently with higher mortality. Adenosine, Enalapril, and Lidocaine are not typically the first line of treatment in these cases, with Lidocaine being more used for ventricular arrhythmias, Enalapril as an ACE inhibtor is for blood pressure control, and Adenosine mainly for diagnosing and treating supraventricular tachycardias. RESPONSE: Thus, based on the patient's AB refibrillation, his initial responsive to Metoprolol and the drug's overall suitability for the condition, I believe another dose of Metoprolol (choice E) should be administered intravenously to decrease the patient's pulse rate.

**AI_C:** Restating the Question: The presented scenario is a medical case in which a 47-year-old male patient was admitted to the hospital with sudden symptoms of palpitations, left-sided chest pain, light-headedness, and shortness of breath. His medical history includes paroxysmal atrial fibrillation, a condition characterized by irregular heart rhythm causing problems with efficient blood flow. On admission, his pulse was extremely high at 146/min. He was given oxygen and metoprolol, a drug often used to control fast heart rates in atrial fibrillation. After his pulse decreased initially, it has since risen again to an elevated level of 160/min with a maintained blood pressure. The question is seeking to determine the most appropriate intravenous medication that should be administered to decrease the patient's pulse further. Planning: The assumption in this question is that the patient's rise in pulse rate after initial treatment with metoprolol indicates a need for additional or different pharmacological intervention. This raises the question of whether or not the prior treatment was effective, and if it wasn't, why that might be the case. A possible alternate assumption is that the initial treatment with metoprolol was effective but perhaps temporarily due to the paroxysmal nature of his atrial fibrillation. If the assumption is correct that additional or different pharmacological intervention is necessary, the next step would be to evaluate the best drug of choice. Clinical Reasoning: When considering the appropriate pharmacological treatment, it is essential to take into account the pathophysiology of atrial fibrillation. This condition is characterized by irregular and often rapid heart rate. The primary therapeutic goal is usually to control the heart rate and rhythm and to prevent thromboembolic complications. Various drugs may be effective depending on the specifics of the case, and it's important to consider the patient's past and current response to metoprolol, his overall cardiac health, and any potential risk factors for other treatment options. Concluding and Response: Therefore, given the fact that metoprolol had an initial positive effect on this patient by reducing his pulse from 146/min to 90/min, re-administration of metoprolol should be considered as the next step. Metoprolol is a beta-blocker that can reduce the heart rate, lower blood pressure, and decrease the heart's output of blood, thereby reducing its workload. Its effectiveness in treating this case has already been demonstrated by the patient's initial response. The return to tachycardia could simply be a reflection of the paroxysmal nature of the patient's atrial fibrillation, rather than a failure of the treatment method. Consequently, my answer would be (E) Metoprolol.

**AI_B:** Restatement: We're asked to suggest a treatment course for a 47-year-old man with a medical history of paroxysmal atrial fibrillation, currently hospitalized due to sudden palpitations, chest pain, light-headedness, and breathlessness. The patient initially responded well to Metoprolol, but his pulse has since spiked. Our goal is to decide which medication would be suitable to administer intravenously to bring the patient's pulse back under control. Planning: The options presented are Adenosine, Digoxin, Enalapril, Lidocaine, and Metoprolol. Adenosine is primarily used for symptomatic supraventricular tachycardia, which isn't the case here. Meanwhile, Lidocaine is used for ventricular arrhythmias, which also doesn't apply to the current patient. Enalapril is an angiotensin-converting enzyme (ACE) inhibitor, used mostly for hypertension and heart failure, which again, doesn't fit the patient's condition. Metoprolol and Digoxin are both used for rate control in atrial fibrillation. The patient initially responded well to Metoprolol, but later his heart rate increased again. Given the sudden increase in heart rate, despite initial response to Metoprolol, we might need to consider other possibilities such as Digoxin. Clinical Reasoning: Metoprolol is a beta-blocker that works by blocking beta-receptors in the heart, preventing the action of adrenaline and noradrenaline, thereby reducing the heart rate. Given the patient's history of atrial fibrillation, using a beta-blocker was an appropriate initial step. However, the sudden elevation of the patient's pulse rate after first responding to Metoprolol may indicate drug resistance or an inadequate dosage. On the other hand, Digoxin slows the heart rate by inhibiting the sodium-potassium ATPase pump, thereby increasing vagal tone and slowing the AV node conduction. It could be an alternative when beta-blockers are not sufficient. Conclusion: Given the patient's re-elevated heart rate after initially responding to Metoprolol, there could be an argument for switching to Digoxin. However, it's also possible that the initial dose of Metoprolol was insufficient for this patient. The patient's response to Metoprolol indicates that his body responds to the pharmacologic action of the medicine. So, it might be beneficial to act on the finding by adjusting the dosage. Response: Therefore, I propose the Metoprolol (Option E), with careful monitoring of the patient's response. If the patient's heart rate remains uncontrolled, a reevaluation should be done considering other medications like Digoxin. This not only provides an effective heart rate control strategy but also offers a more personalized approach addressing each patient's unique medical situation and reaction to the drug.

**AI_A:** Restating the question, a 47-year-old man known with paroxysmal atrial fibrillation comes in with palpitations, chest pain, light-headedness, and shortness of breath. Despite initial administration of intravenous metoprolol which dropped his heart rate from 146/min to 90/min, his pulse shot back up to 160/min within two hours of admission. He's now looking for a drug that can reign in his rapid heartbeat yet again. So, here's our plan. We have a list of five drugs to consider: Adenosine, Digoxin, Enalapril, Lidocaine, and Metoprolol. They all act differently and some aren't typically used for this situation. Our priority right now is to identify the best drug that can lower the patient's heart rate effectively and safely. Let's start clinically reasoning this. Adenosine is typically used to treat supraventricular tachycardia (SVT) and is unlikely to be efficacious in atrial fibrillation. Enalapril, being an ACE inhibitor, primarily acts on blood pressure and not primarily on heart rate control. Lidocaine is an antiarrhythmic primarily used in ventricular arrhythmias, not atrial fibrillation. Digoxin and Metoprolol remain our potential candidates. Both drugs have been used in the management of atrial fibrillation to control ventricular rate. Digoxin works by increasing vagal tone which slows conduction at the AV node. Metoprolol is a beta blocker that decreases AV nodal conduction. The patient’s initial response to metoprolol suggests that the drug is effective in controlling his heart rate initially. His later increase in rate may represent the paroxysmal nature of his condition or transient loss of control. Given these findings, we can draw a conclusion. Considering the safety profile and the patient's initial positive response, Metoprolol would be a logical choice in trying to regain rate control. So given the way these drugs work and how the patient responded, the correct answer would be (E) Metoprolol. Metoprolol is a beta-blocker used primarily in rate control for atrial fibrillation. It's safe, effective, and the patient responded well to it initially. A second dose may be needed due to the paroxysmal nature of this patient's condition. Patient's such as these may benefit from additional or higher doses than initially used, and while more chronic management may eventually include other medications such as Digoxin (B), in an acute setting like this with demonstrated initial response, metoprolol is a sensible choice to try and regain rate control.

**Facilitator** The consensus among the AI responses is that the most appropriate course of action for the 47-year-old man with paroxysmal atrial fibrillation, who initially responded to Metoprolol but then experienced a spike in heart rate, is to administer another dose of Metoprolol (E). The reasoning behind this consensus is based on the patient's initial positive response to Metoprolol, a beta-blocker that effectively reduces heart rate by blocking the action of adrenaline and noradrenaline. The subsequent increase in heart rate could be attributed to the paroxysmal nature of the patient's condition, rather than a failure of the drug. While other drugs like Digoxin, Adenosine, Enalapril, and Lidocaine were considered, they were deemed less suitable. Digoxin, although effective in controlling ventricular rate in atrial fibrillation, works more slowly and might not be as effective in an acute setting. Adenosine is typically used for supraventricular tachycardia and may not be efficacious in this case. Enalapril, an ACE inhibitor, primarily acts on blood pressure rather than heart rate control. Lidocaine is primarily used in ventricular arrhythmias, not atrial fibrillation. Therefore, given the patient's initial positive response to Metoprolol and its known efficacy in controlling heart rate in atrial fibrillation, a second dose of Metoprolol is recommended. However, careful monitoring of the patient's response is necessary, and if the heart rate remains uncontrolled, a reevaluation should be done considering other medications like Digoxin.
